# Persistence of live virus in critically ill patients infected with SARS-COV-2: a prospective observational study

**DOI:** 10.1186/s13054-021-03884-z

**Published:** 2022-01-04

**Authors:** Duane J. Funk, Jared Bullard, Sylvan Lother, Gloria Vazquez Grande, Lauren Garnett, Kaylie Doan, Kerry Dust, Anand Kumar, Guillaume Poliquin, Jim Strong

**Affiliations:** 1 Departments of Anesthesiology and Medicine, Section of Critical Care, Max Rady College of Medicine, University of Manitoba, 2nd Floor Harry Medovy House, 671 William Avenue, Winnipeg, MB R3E 0Z2 Canada; 2 Cadham Provincial Laboratory, Manitoba Health, Winnipeg, MB Canada; 3 Department of Pediatrics and Child Health, University of Manitoba, Winnipeg, MB Canada; 4 Department of Medical Microbiology and Infectious Diseases, University of Manitoba, Winnipeg, MB Canada; 5 National Microbiology Laboratory, Public Health Agency of Canada, Winnipeg, MB Canada; 6 Department of Medicine, Sections of Infectious Disease and Critical Care, University of Manitoba, Winnipeg, MB Canada

**Keywords:** COVID-19, Viral culture, RT-PCR, Infection control

## Abstract

**Background:**

Research on the duration of infectivity of ICU patients with COVID-19 has been sparse. Tests based on Reverse Transcriptase polymerase chain reaction (RT-PCR) detect both live virus and non-infectious viral RNA. We aimed to determine the duration of infectiousness based on viral culture of nasopharyngeal samples of patients with COVID-19.

**Methods:**

Prospective observational study in adult intensive care units with a diagnosis of COVID-19 Pneumonia. Patients had repeated nasopharyngeal sampling performed after day 10 of ICU admission. Culture positive rate (based on viral culture on Vero cells in a level 4 lab) and Cycle threshold from RT-PCR were measured.

**Results:**

Nine patients of the 108 samples (8.3%, 95% CI 3.9–15.2%) grew live virus at a median of 13 days (interquartile range 11–19) after their initial positive test. 74.1% of patients were RT-PCR positive but culture negative, and the remaining (17.6%) were RT-PCR and culture negative. Cycle threshold showed excellent ability to predict the presence of live virus, with a Ct < 25 with an AUC of 0.90 (95% CI 0.83–0.97, *p* < 0.001). The specificity of a Ct > 25 to predict negative viral culture was 100% (95% CI 70–100%).

**Conclusion:**

8.3% of our ICU patients with COVID-19 grew live virus at a median of 13 days post-initial positive RT-PCR test. Severity of illness, use of mechanical ventilation, and time between tests did not predict the presence of live virus. Cycle threshold of > 25 had the best ability to determine the lack of live virus in these patents.

## Introduction

The COVID-19 pandemic has strained health care resources throughout the world. The last line of defense, and the most scarce resource in the developing world, is the number of acute care or intensive care unit (ICU) beds able to care for patients infected with SARS-CoV-2. In addition to the labor-intensive support, these patients require the need for isolation rooms and for health care workers to don and doff personal protective equipment in order to prevent nosocomial infection. To date, research on the duration of infectivity of ICU patients with COVID-19 has been sparse. This has led to divergent guidelines with respect to the discontinuation of isolation precautions in patients with COVID-19. Currently, the CDC recommends a symptom-based approach of 10 days of isolation as the likelihood of detecting replication competent virus after this time period approached zero [1–3].

Unknown is if the duration of live viral shedding differs in the critically ill. Several studies have looked at viral loads in hospitalized patients and have found a correlation with disease severity [4–6]. These studies used Reverse Transcriptase polymerase chain reaction (RT-PCR) results as a surrogate for live virus. A major drawback to RT-PCR and other diagnostic approaches is that they all fail to determine virus infectivity: RT-PCR sensitivity is excellent but specificity for detecting replicative virus is poor. We have shown that viral RNA can persist beyond infectivity [3, 7]. As a result, demonstration of in vitro infectiousness on cell lines is a more informative surrogate of viral transmission. The ability of viral culture to inform infectivity is an important aspect of diagnostics but its use is hampered by its difficult and labor-intensive nature.

The aim of our study was to determine the duration of infectiousness of critically ill patients with SARS-CoV-2 as determined by cell culture positivity.

## Materials and methods

### Ethics approval

The study was performed in accordance with protocol HS23906 (H2020:211) and approved by the University of Manitoba Research Ethics Board. The REB waived the need for informed consent as samples were obtained as part of routine clinical infection control practices and public health management and were not taken specifically to be included in the current study.

### Patients

We enrolled adult patients over 18 years of age admitted to our tertiary care academic Intensive Care Units (ICUs) with the diagnosis of COVID-19 Acute Respiratory Distress Syndrome, that was diagnosed according to the Berlin criteria and had a positive RT-PCR test for the E gene of the SARS-CoV-2 virus [8]. Symptom duration was not recorded as during this wave of COVID in our province as lockdown measures were instituted by the Public Officer of Health, and as such, patients were often disingenuous with symptom duration.

As part of the standard admission order set in our hospital, all patients received dexamethasone and tocilizumab for their COVID-19 ARDS. Patients also received ceftriaxone and azithromycin if there was a concern of a superimposed bacterial pneumonia. None of our patients received Remdesivir or convalescent plasma.

All the infections were acquired in the community, and none of the infections were of a nosocomial source. Levels of inflammatory biomarkers and COVID-ARDS phenotype determination are not performed at our institution and are therefore not recorded.

At the time of this study, the alpha variant was the predominant variant in our Province with 84% of our cases being alpha, 5% delta, and 6% undetermined lineage.

### SARS-CoV-2 RT-PCR cycle threshold values

Through public health, epidemiology/surveillance and laboratory records, date of first positive test was determined. Repeated nasopharyngeal sampling was obtained greater than 9 days after the initial positive test. We chose greater than 9 days as a minimum as we felt this would allow sufficient time for the immune response to render any live virus inactive. For all positive samples, the SARS-CoV-2 envelope (E gene) RT-PCR Ct values were obtained. Contemporaneous bronchial washing sampling was not performed.

RT-PCR testing was performed by Cadham Provincial Laboratory (CPL), the reference laboratory for SARS-CoV-2 testing for the Province of Manitoba. All samples were tested using laboratory developed testing (LDT) to minimize Ct variation. Specimens were collected in the ICUs and transported in viral transport medium (VTM) to CPL. In the laboratory, the specimens were stored at 4 °C for 24 h until they were tested as previously described [3].

### Tissue culture infectious dose 50% (TCID50) cell culture assay

Vero cells (ATCC: CCL-81) were grown in sterile tissue culture flasks with vented caps in modified Eagle’s medium (MEM) supplemented with 5% fetal bovine serum (FBS), 1% penicillin/streptomycin, 0.5 μg/mL amphotericin B, and 1% L-glutamine and maintained in a 37 °C incubator with 5% CO2. For TCID50 testing, cells were seeded into 96-well plates (Thermo Scientific, 167,008) at ~ 70% confluency. Using dilution blocks, patient samples were serially diluted tenfold from 10^−1^ to 10^−8^ in MEM supplemented with 2% FBS, 1% penicillin/streptomycin, 0.5 μg/mL amphotericin B, and 1% L-glutamine. Dilutions were placed onto the Vero cells in triplicate and incubated at 37 °C with 5% CO2 for 96 to 120 h for subsequent assessment of cytopathic effects and TCID50 reading. Mock infected controls served as comparator.

### Generation of log copies per milliliter SARS-CoV-2 RNA standard curve

All experiments with live SARS-CoV-2 were performed in a BSL4 laboratory. SARS-CoV-2 stock was serially diluted threefold from 3.33^–1^ to 3.18^–11^ in VTM. To remove samples from BSL4 for further analysis, 140 µl of sample was inactivated in 560 µl Buffer AVL for 10 min and then the contents were transferred to a tube containing 560 µl 100% ethanol for an additional 10 min. RNA was extracted from samples using QIAmp viral RNA Minikit (QIAGEN, Valencia, CA) following manufacturer’s instructions. MS2 phage was spiked into the AVL as an exogenous PCR control such that 560 μL of AVL contains 500 pfu of MS2 phage (~ 50,000 RNA copies—data not shown).

Additionally, to calculate genome copies from a standard curve, SARS-CoV-2 stock virus was inactivated, and RNA was extracted viaQIAmp viral RNA Minikit (QIAGEN, Valencia, CA) following manufacturer’s instructions. Extracted RNA was then serially diluted tenfold in TE buffer to produce the standard curve (data not shown) and quantified against synthetic RNA (BEI Resources) samples that were similarly serially diluted from the original 4.82E7 genome equivalents/mL. The viral stock standard curve was completed with all RT-qPCR runs in order to equivocate Ct value(s) at any given quantity of dilution to genome copies/mL.

### Clinical and laboratory data

Baseline demographic data including age, gender, date of original test, baseline medical comorbidities (diabetes mellitus, hypertension, obesity, chronic kidney disease, coronary artery disease, underlying respiratory disease, cancer, current immunosuppressed state, and pregnancy) were abstracted from the medical record. Laboratory, hemodynamic and respiratory values required to calculate the Sepsis related Organ Failure Assessment (SOFA) Score were also collected at admission to the ICU [9]. RT-PCR values for human RNAse P gene, an endogenous internal amplification control, were used as a marker of quality of the nasopharyngeal sample.

### Statistical analysis

From our previous work, we knew that adults had a culture positive rate of 28.9% and that culture positivity declined substantially from time of symptom onset. As such, we hypothesized that ICU patients would have a 75% reduction in culture positive rates at greater than or 9 days. In order to confirm this, with power 0.8 and two sided alpha at 0.05 would require 101 ICU patients.

Data are presented as mean ± standard deviation for normally distributed data and as median [Interquartile range] for non-normally distributed data. *p* values are reported as two tailed. Between-group comparisons were performed using a Student’s *t* test or Mann–Whitney test for continuous variables. Categorical data were analyzed using Fisher’s exact test. Kruskal–Wallis ANOVA was used for comparison of the nonparametric group medians with Dunn’s correction for multiple comparisons. Normality was assessed using the Kolmorgorov–Smirnov test, and logistic regression was performed with robust standard errors. *p* values less than 0.05 were considered significant.

Statistical analysis was performed with Stata V16.1 (College Station, Texas, USA), and GraphPad Prism 9 (San Diego, CA, USA).

## Results

Results for the 108 patients studied (divided into those that were culture positive vs. those that were culture negative) are presented in Table 1. Seventy-two percent of the patients required invasive mechanical ventilation, and 26% required high flow nasal cannulae, the remainder required supplemental oxygen via nasal prongs or face mask.

**Table 1:** Baseline characteristics of patients who were and were not culture positive for SARS-CoV-2

Characteristic	Culture positive *N* = 9	Culture negative *N* = 99	*p* Value
Age	61 ± 18	60 ± 16	0.87
Male sex number (%)	4 (40%)	46 (46%)	0.70
Days between tests	13 [11–19]	14 [12–16]	0.39
Cycle threshold	19 [17.5–22.5]	29 [25–32]	< 0.001
Log Copies RNA	7.4 [6.3–7.9]	4.4 [3.5–6.0]	< 0.001
RNAseP	26 ± 2.3	26 ± 2.3	0.63
Mechanical ventilation	6 (66%)	72 (72%)	0.70
Diabetes	3 (33%)	53 (53%)	0.13
Hypertension	2 (22%)	52 (52%)	0.11
Chronic kidney disease	1 (11%)	22 (22%)	0.34
Coronary artery disease	3 (33%)	18 (18%)	0.12
Obesity	5 (55%)	35 (35%)	0.24
Respiratory disease	0 (0%)	24 (24%)	0.21
Cancer	1 (11%)	6 (6%)	0.32
Immunosuppressed	2 (22%)	14 (14%)	0.32
SOFA Score	6 [6, 7]]	7 [6–8]	0.14

**Fig. 1:**
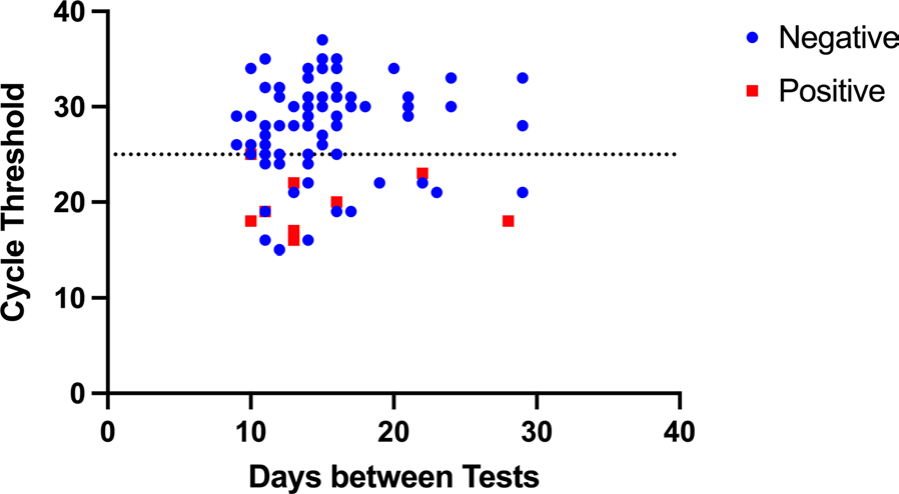
Graph demonstrating cycle threshold (Ct) vs. days between tests. There was no relationship between the ability to culture live virus and the number of days between tests. No sample grew live virus with a Ct > 25. Samples that were culture negative are in blue, while those that were positive are red

Nine patients of the 108 studied were culture positive (8% 95% CI 4–15%). All these patients were also PCR positive. Eighty patients (74% 95% CI 65–82%) were RT-PCR positive but culture negative. Nineteen patients (18%, 95% CI 11–26%) were RT-PCR negative for SARS-CoV-2. This is graphically represented in Fig. 1. Baseline characteristics of the patients who were culture positive and culture negative are presented in Table 1. There was no difference in the mean age between culture positive and negative patients (61 (18) vs. 60 (16) years, *p* = 0.87). There was also no difference in male sex, or the need for mechanical ventilation between the culture positive and culture negative groups. There was also no difference in baseline comorbidities between the groups.

Median days between tests for the patients who were RT-PCR negative (14 [13–21]), RT-PCR positive but culture negative (14 [–16]), and RT-PCR and culture positive (13 [11–19]) were not different between the groups (*p* = 0.39, Kruskal–Wallis ANOVA).

**Fig. 2:**
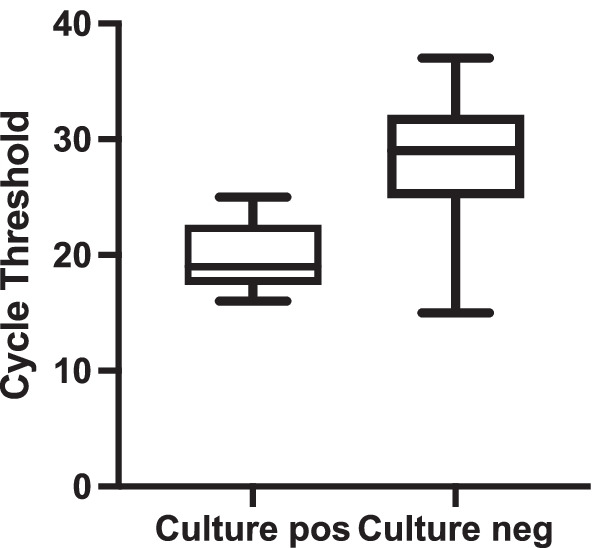
Comparison of cycle threshold between patients that were culture positive vs. culture negative. Patients who had live virus cultured had a significantly lower Ct than those where live viruses could not be cultured (19 [18–23] vs. 29 [25–32] respectively, *p* < 0.001). Line is at the median, and the box edges represent the interquartile range. Arrows at the end of the boxes reflect the 95% range of values

The cycle threshold was significantly lower in the culture positive vs. the culture negative group (Fig. 2; 19.8 ± 3.0 vs. 28.0 ± 5.1, respectively, *p* < 0.001), and this was also reflected in higher Log copies RNA/ml between the two groups (7.4 [6.3–7.9] vs. 4.4 [3.5–6.0] culture positive vs. culture negative, respectively, *p* < 0.001). Cycle threshold is a semi-quantitative measure of how much genetic material is present in the initial sample. If more RT-PCR cycles are required to detect SARS-CoV-2, then less viral RNA was present in the sample.

Ct values for human RNAse P gene, an endogenous internal amplification control used as a marker of quality of the nasopharyngeal sample, were not different between the culture positive and culture negative groups (26 (2.3) vs. 26 (2.3), *p* = 0.63). This suggests that the difference in culture positive results and Log RNA levels was not due to differences in sample quality.

**Fig. 3:**
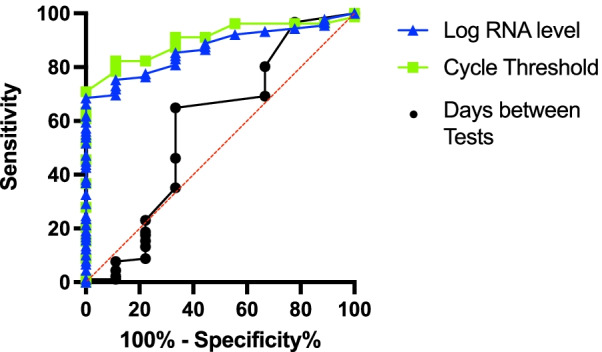
Receiver operating characteristic curves for cycle threshold (Ct), Log RNA Copies/mL and days between tests. The AUC for Ct was 0.90 (95% CI 0.83–0.97, *p* < 0.001), for Log Copies RNA/ml: 0.87 (95% CI 0.79–0.95, *p* < 0.001) and for time between tests the AUC was 0.58 (95% CI 0.35–0.82, *p* = 0.41). The AUC for Ct and days between tests was significantly different (*p* = 0.01). The specificity of a Ct > 25 to predict negative viral culture was 100% (95% CI 70–100%)

**Fig. 4:**
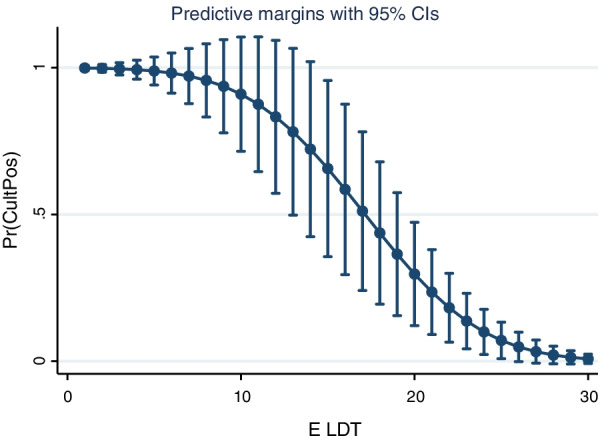
Probit regression of the probability of being culture positive and the cycle threshold value of RT-PCR of the SARS-CoV-2 E gene

Receiver operating characteristic curve analysis of the Ct to discriminate between patients with and without positive viral culture showed an AUC of 0.90 (Fig. 3: 95% CI 0.83–0.97, *p* < 0.001). Similarly, the ROC analysis of Log Copies RNA/ml to determine culture positivity also showed good predictive ability with an AUC of 0.87 (95% CI 0.79–0.95, *p* < 0.001). ROC analysis of the time between tests was not able to discriminate between those patients who were culture positive and culture negative (AUC 0.58, 95% CI 0.35–0.82, *p* = 0.41). The area under the ROC curves for Ct and days between tests was significantly different (*p* = 0.01). The specificity of a Ct > 25 to predict negative viral culture was 100% (95% CI 70–100%). Probit regression analysis demonstrated that there would be a less than 2.5% probability of retrieving live virus from a patient with a Ct > 25 (Fig. 4).

The TCID50/mL for patients who were culture positive was 562 [178–3160]. Fifty percent tissue culture infective dose (TCID50) is a measure of infectious virus titer and represents the amount of virus required to kill 50% of cells in inoculated tissue culture.

Multivariable logistic regression using culture positivity as the outcome variable, and cycle threshold, SOFA score, and the need for mechanical ventilation as predictor variables demonstrated cycle threshold as the only parameter that was predictive of culture positivity. The odds ratio for Ct was 0.71 (95% CI 0.59–0.87, *p* = 0.001). This implies that for every one unit increase in Ct, there was a 29% decrease in the odds of being culture positive.

To determine if there was an association between days after initial positive PCR testing and the odds of being culture positive, we added days between PCR tests as a continuous variable to our logistic regression model and found that the odds ratio was 1.1 (95% CI 0.87–1.37, *p* = 0.44). This would suggest that days between tests were not strongly associated with being culture positive.

## Discussion

Our study demonstrated that in critically ill patients with COVID-19, almost 10% of patients continued to shed replication competent virus after 10 days. The median duration of shedding of live virus among the these patients was 13 [11–19] days post-initial testing. Almost three quarters of patients were still RT-PCR positive at a median of 14 [12–16] days after an initial positive test. The remaining 17.6% patients were RT-PCR negative for SARS-CoV-2 at 14 [12–16] days post-initial testing.

There was no difference in baseline medical comorbidities between the culture positive and culture negative groups. There was also no difference in the median number of days from a patient’s initial positive test, and their ability to grow replication competent virus upon repeat testing.

The TCID50/mL for culture positive samples was 562. Of note, this was not significantly lower than the median value that we had previously reported in a study of outpatients with COVID-19 (1780 [282–8511] vs. 562 [178–3160], *p* = 0.99) despite the ICU patients being sampled further into their illness (3 [2–4] vs. 13 [11–19] days, respectively, *p* < 0.001]. While the exact infectious dose of SARS-CoV-2 is not known, it is estimated to be between 36 and 179 virions, meaning that our value of 562 would theoretically be enough to cause infection [10].

Other groups have attempted to describe the relationship between RT-PCR positive and culture positive patients. Singanayagam et. al looked at a subset of 20 patients with critical illness in their larger study of 253 patients with COVID-19 and found that the median Ct of culture positive samples in this group was higher than ours (32.6 [28.4–33.4]) [11]. The authors did not report their culture positive rate of this subset of patients, and they used SARS-CoV-2 RNA-dependent RNA polymerase as their target, making comparisons to our results difficult.

Cui et al. took samples from 21 critically ill patients and found that they were unable to grow live virus if the Ct value was greater than 28.4, and they had no positive viral cultures after 12 days in hospital [12]. This group utilized the N gene of SARS-CoV-2, and only one of the patients was mechanically ventilated, and the median SOFA score was 0, suggesting that their patients were less ill than our patients.

The largest study to date looking at the duration of viral shedding was by van Kampen et al. who looked at the ability to recover replication competent virus in a group of 129 hospitalized patients [5]. Eighty-nine of these patients were in ICU, and 91% of them were receiving mechanical ventilation. The authors did not report SOFA or other severity of illness scores, but presumably, with the high rates of mechanical ventilation, their patients were similar to ours. These authors found that the ability to recover live virus was statistically unlikely when the viral load was below 6.63 Log_10_ RNA copies/mL. As these authors used the same target gene as we did (E gene), this value corresponds to a Ct of ~ 22 which is consistent with our data. The slight variation is likely due to differences in the standard curve creation of Ct and Log_10_ RNA copies/mL.

Multivariable logistic regression, using culture positivity as a dependent variable and SOFA score, need for mechanical ventilation and RT-PCR cycle threshold demonstrated that only cycle threshold was predictive of a positive viral culture on repeat testing (OR 0.71, 95% CI 0.59–0.87, *p* = 0.001). This implies that for every one unit increase  in Ct, there was a 29% decrease in the odds of being culture positive. Further, ROC analysis demonstrated that a Ct > 25 was highly predictive of not being able to recover live virus with a specificity of 100% (95% CI 70–100%). This also suggests that patient factors are not helpful in determining culture positivity, but this result is limited by the small number of positive culture results in our study.

Our results have implications for the de-escalation in isolation precautions for patients with COVID-19 in ICUs. There are a variety of policies that have been advocated for de-escalation of precautions in patients with COVID-19 [1, 2]. These include a time based or symptom-based strategy, or a strategy based on repeat testing. Our results suggest that duration between tests nor symptoms nor severity of illness can predict the presence of replication competent virus. In fact, there were two patients who remained culture positive greater than 20 days after their initial test. Patients can remain RT-PCR positive for a significant length of time after their initial test, and not grow live virus. The longest length of time a patient was RT-PCR positive was 29 days. This results in prolonged isolation requirements, and the need for more personal protective equipment use by heath care providers in patients who are not infectious. The CDC currently recommends a longer isolation period for critically ill patients with COVID-19 [1]. Our results suggest that in some cases, this time may be excessive, and in others, insufficient to ensure the absence of live virus.

PCR and other nucleic amplification (NA) strategies have surpassed viral culture as the gold standard viral diagnostic, because of their wider application, higher sensitivity, rapid performance, and ability for field deployment. A major drawback to PCR and other diagnostic approaches (including other NA, serology, antigen detection) is that they all fail to determine virus infectivity: PCR sensitivity is excellent but specificity for detecting replicative virus is poor [13].

Cycle threshold, an easily obtainable number from RT-PCR results, had excellent ability to predict the presence of live virus. A de-escalation strategy of isolation precautions that involves re-testing critically ill patients and, if patients are RT-PCR positive with a Ct > 25, isolation precautions can be safely removed with a low risk of remaining infectious. The Ct threshold of 25 for detection of live virus in ICU patients is remarkably consistent with previous work we have done in outpatient adults and pediatric patients with COVID [3, 7].

Limitations to our study include the limited sample size (although the largest to date), and the lack of repeat testing to determine when patients transitioned to a negative culture/RT-PCR test. We felt that daily repeated tests in this patient population were overly invasive, considering how long patients may remain RT-PCR positive. Degradation of sample from collection to viral culture, due to a freeze–thaw cycle, also may have reduced our yield. However, even if this was case, the consistency of the Ct in this study, our previous work and that of van Kampen strongly suggests that the Ct can be used to predict the presence of live virus and may play a role in discontinuation of isolation precautions in patients with COVID-19 in the ICU. Our study did not look at lower respiratory tract specimens, leaving the possibility that the lower respiratory tract could still contain live virus. The discordance between upper and lower respiratory tract sample positivity is possible and has been demonstrated in other studies [14, 15]. The number of immunosuppressed patients in our study is small, and it is well known that this group can shed virus for a prolonged period. We are therefore not able to examine risk in this population due to small sample size.

Institutions that use other qRT-PCR assays and PCR targets will need to determine the threshold for live viral growth based on cycle threshold, thereby potentially limiting the applicability. Previous work has shown that the cross-platform validity for PCR of the E gene of SARS-CoV-2 is robust, suggesting that the Ct from different platforms for this gene may be used to predict the presence of live virus [16]. The salient point, however, is that PCR detects both culturable, and non-culturable/non-infectious viral particles, and that the Ct cutoff can be defined above which no live virus can be found.

Finally, the sampling was done before the delta strain of SARS-CoV-2 became dominant in the province. Ongoing work at our institution has shown that it is extremely unlikely to grow live delta variant virus in culture from outpatient samples when the sample has a Ct greater than 25. This is consistent with previous work we have completed in outpatients who grew wild type virus, so we feel it is likely that the Ct value will hold true for variant infections in the ICU population [3, 7].

## Conclusion

In conclusion, 8% (95% CI 4–15%) of our ICU patients with COVID-19 grew live virus at a median of 13 days post-initial positive RT-PCR test. Severity of illness, use of mechanical ventilation, and time between tests were not able to accurately predict the presence of live virus. Cycle threshold of > 25 for the E gene had the best ability to determine the lack of live virus in these patents. A strategy of repeat RT-PCR testing and the use of the Ct to guide discontinuation of isolation precautions can be safely implemented to reduce resource consumption and improve patient care.

## Data Availability

Due to institutional ethics rules, raw data are not available for public disclosure.
